# Novel capsaicin-induced parameters of microcirculation in migraine patients revealed by imaging photoplethysmography

**DOI:** 10.1186/s10194-018-0872-0

**Published:** 2018-06-18

**Authors:** Alexei A. Kamshilin, Maxim A. Volynsky, Olga Khayrutdinova, Dilyara Nurkhametova, Laura Babayan, Alexander V. Amelin, Oleg V. Mamontov, Rashid Giniatullin

**Affiliations:** 1 Department of Computer Photonics and Videomatics, ITMO University, St. Petersburg, Russia; 2 Department of Neurology and Rehabilitation, Kazan State Medical University, Kazan, Russia; 3 Laboratory of Neurobiology, Kazan Federal University, Kazan, Russia; 4 Department of Neurobiology, University of Eastern Finland, Kuopio, Finland; 5 Department of Neurology and Neurosurgery, Pavlov First Saint Petersburg State Medical University, St. Petersburg, Russia; 6 Department of Circulation Physiology, Almazov National Medical Research Centre, St. Petersburg, Russia

**Keywords:** Migraine, Capsaicin, Microcirculation, CGRP, TRPV1, Dermal blood flow, Imaging photoplethysmography

## Abstract

**Background:**

The non-invasive biomarkers of migraine can help to develop the personalized medication of this disorder. In testing of the antimigraine drugs the capsaicin-induced skin redness with activated TRPV1 receptors in sensory neurons associated with the release of the migraine mediator CGRP has already been widely used.

**Methods:**

Fourteen migraine patients (mean age 34.6 ± 10.2 years) and 14 healthy volunteers (mean age 29.9 ± 9.7 years) participated in the experiment. A new arrangement of imaging photoplethysmography recently developed by us was used here to discover novel sensitive parameters of dermal blood flow during capsaicin applications in migraine patients.

**Results:**

Blood pulsation amplitude (BPA) observed as optical-intensity waveform varying synchronously with heartbeat was used for detailed exploration of microcirculatory perfusion induced by capsicum patch application. The BPA signals, once having appeared after certain latent period, were progressively rising until being saturated. Capsaicin-induced high BPA areas were distributed unevenly under the patch, forming “hot spots.” Interestingly the hot spots were much more variable in migraine patients than in the control group. In contrast to BPA, a slow component of waveforms related to the skin redness changed significantly less than BPA highlighting the latter parameter as the potential sensitive biomarker of capsaicin-induced activation of the blood flow. Thus, in migraine patients, there is a non-uniform (both in space and in time) reaction to capsaicin, resulting in highly variable openings of skin capillaries.

**Conclusion:**

BPA dynamics measured by imaging photoplethysmography could serve as a novel sensitive non-invasive biomarker of migraine-associated changes in microcirculation.

## Background

One of the main trends in migraine studies is to find out the most sensitive and preferably non-invasive biomarkers serving for the diagnostic and personalized treatments of this often-intractable disorder. Application of capsaicin to the skin is widely used to monitor the reactiveness of local blood flow following activation of capsaicin-sensitive TRPV1 receptors [1, 2]. The underlying mechanism of redness (flare) is mainly associated with the release of the neuropeptide CGRP from nociceptive C-fibers expressing TRPV1 receptors in membrane [3]. Several recent studies in animals and humans suggested the role of TRPV1 receptors in migraine [4, 5]. Accumulated evidence also suggested that CGRP is the main neuropeptide implicated in migraine pathology [6–8]. Many modern approaches for migraine treatment are based on inhibition of CGRP driven pro-nociceptive activity [7, 9]. This requires simple tests to evaluate the release of CGRP in humans. Thus, capsaicin-induced increase in dermal blood flow (DBF) is widely used to test the activity of potential anti-migraine medicines [2]. Apart from general pharmaceutical significance, this DBF method could be attractive to develop personalized approaches for migraine treatments, for instance, to test the sensitivity of the particular individual to capsaicin as a prognostic of CGRP mediated component in his/her vascular reactions. In this regard, it is worth noting that people with TRPV1 rs8065080 polymorphism are differentially sensitive to capsaicin [1] providing a genetic background for the distinct reactivity.

A recent study has shown that migraine patients reported higher level of capsaicin-induced feeling of pain and larger areas of flare [10]. In most studies simple measurement of the flare size is commonly used as the index of activation of capsaicin receptors [4, 10] although it is clear that this general reaction is determined by many non-specific factors such as the thickness of skin, its native color and, importantly, the level of CGRP released from the skin nerves [2].

Laser Doppler imaging systems are commonly used for assessment of the cutaneous microcirculation [11, 12]. However, these systems provide sequential scanning of the area under study, which increases the time needed to collect information from the area under study when its size is big and high spatial resolution is required. In contrast, imaging photoplethysmography (IPPG) systems acquire information from multiple points in parallel and simultaneously thus providing high spatial and temporal resolution [13]. It was shown recently that these systems are capable to visualize capillary blood flow [14, 15] making IPPG method more preferable for detailed study of spatial-temporal variations of microcirculation. Previously we suggested the non-invasive IPPG technique to evaluate the vascular reactions in migraine [16, 17]. Recently we advanced this technique by linking peripheral changes in pulsatile blood flow with heart rate, which largely increased the sensitivity of this combined approach [18]. In the current study, we used IPPG to monitor parameters of local blood circulation in the skin during capsaicin applications in patients with migraine and compared those with microcirculation in control group. With this technique we find out highly heterogeneous (both in time and space) reaction of migraine patients to stimulation of TRPV1 receptors that control local microcirculation. We also show that BPA does not always coincide with redness (or skin flare) and can serve as potential biomarker of blood flow changes associated with migraine and probably with other cardiovascular diseases.

## Methods

### General description of participants

The study was conducted in St. Petersburg and Kazan in accordance with ethical standards presented in the 2013 Declaration of Helsinki. The Ethics Committees of the Pavlov First Saint-Petersburg State Medical University and Kazan Federal University prior the research approved the protocol of this study. The study involved 14 patients with migraine and 14 healthy volunteers. Both groups were comparable in age, main constitutional, and hemodynamic parameters (see Table 1). An informed consent was obtained from all participants prior to enrollment.

**Table 1:** Healthy subjects and migraine patients

Parameter	Control group	Migraine patients	Significance of differences
Population	14	14	*p* > 0.05
Female/male	6/6	10/4	*p* > 0.05
Age, years	33.7 ± 9.8	34.6 ± 10.2	*p* > 0.05
Body mass index, kg/m^2^	23.1 ± 2.8	22.5 ± 2.6	*p* > 0.05
Systolic blood pressure, mmHg	122 ± 11	121 ± 12	*p* > 0.05
Diastolic blood pressure, mmHg	77 ± 7	79 ± 7	*p* > 0.05
Heart rate	78 ± 13	74 ± 14	*p* > 0.05

### Patients’ selection

The diagnosis of migraine was established according to the International classification of headache disorder (third edition, beta version, ICHD-III3-beta) [19]. Patients meeting the following criteria were included in the study: previously diagnosed migraine with/without aura (as defined in ICHD-3-beta), age from 18 to 55. Exclusion criteria included chronic diseases in stage of decompensation, oncology, pregnancy, breast feeding, intolerance or allergic reaction to lidocaine or capsaicin, treatment by CGRP neutralizing antibodies or antibodies blocking the function of CGRP receptors in the history. In total 48 patients were evaluated for inclusion and exclusion criteria. Twelve patients were excluded due to oncological diseases, pregnancy or breast feeding. Thirty-six patients met the inclusion criteria. Twenty-two people declined to participate in the study. Fourteen patients and 14 healthy volunteers provided informed consent to take part in the study and had undergone the intervention. Most patients with migraine had experienced an episodic form of disease (Table 2). An aura during an attack occurred in a third of patients. None of the patients used CGRP neutralizing antibodies or antibodies blocking the function of CGRP receptors. The frequency of attacks was varying from one to fifteen per month (4.6 ± 4.1) and disease duration from 6 to 30 years (14.7 ± 9.2). The duration of the attack varied from 24 to 72 h. Six patients used triptans, 10 used NSAIDs, while three patients used both types of medications to reverse symptoms. In all patients, the examinations were conducted during the interictal period, at least one day after the last episode of headaches. A period of ten days minimum had passed since the last use of triptan medication.

**Table 2:** Clinical characteristics of patients with migraine

Index	Value
Form of migraine, rare / frequent / chronic	11 / 2 / 1
Aura, n (%)	4 (28.6%)
Cupping migraine attack by triptans, n (%)	6 (42.9%)
Frequency of attacks, per month	4.6 ± 4.1
Duration of the disease, years, age	14.7 ± 9.2

### Experimental technique

**Fig. 1:**
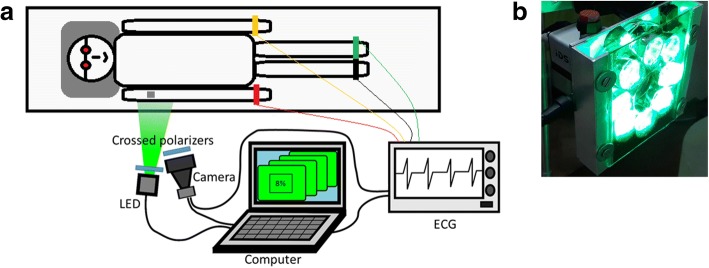
Experimental technique. **a** Layout of the setup for photoplethysmographic image acquisition simultaneously with ECG and **b** photograph of the unit containing digital camera and illuminator with eight green LEDs

Parameters of the cutaneous blood flow were measured at the upper arm of each subject by using imaging photoplethysmography (IPPG) method [18]. Figure 1 shows the schematic presentation of the experimental setup. During the experiment, the subject was laid in horizontal position. His upper arm was illuminated by the green light (at the wavelength of 530 ± 25 nm) generated by eight light-emitted diodes (LEDs) as shown in Fig. 1b. The imaging lens of a digital black-and-white CMOS camera (8-bit model GigE uEye UI-5220SE of the Imaging Development Systems GmbH) was situated in the central part of the LEDs ring. Cross polarizing films were attached ahead of the lens and LEDs to eliminate superficial reflection from the skin [20]. All images were recorded at the frame rate of 39 frames per second with the resolution of 752 × 480 pixels, and transferred frame-by-frame in the PNG format into a personal computer for subsequent off-line analysis. An electrocardiogram (ECG) was recorded simultaneously and synchronously with video frames to improve the quality of data processing [18]. All video recordings were carried out in a dark laboratory room at the temperature of 23 ± 1 °C whereas subject’s eyes were protected by special glasses that do not transmit the green light.

### Protocol of the study

The experimental protocol included the following steps.

1. Before measurements, each subject was asked to relax in the laboratory during 15 min. After relaxation, his/her blood pressure was measured. Thereafter, subject took the recumbent position for video recording of an area of the upper arm during 20 s by means of the IPPG system (Fig. 1). This recording was used to estimate the baseline of blood circulation parameters.
2. In the next step, local anesthesia with 10% lidocaine was applied in an area on the upper arm for 1 h by using gauze patch. During this period, the subject was free in movements. After removal of the lidocaine patch, the subject was asked to take recumbent position again for the recording of 20-s video using IPPG system.
3. Then the patch (5 × 5 cm^2^) containing 8% capsaicin (officinal Quatenza patch) was applied to the upper-arm area recorded by the IPPG system in the previous steps. Application of the Quatenza patch lasted from 15 to 25 min during which the subject was asked to keep the recumbent position and refrain from movements. Video images of the area with Quatenza patch were recorded during 20 s repeating the recording about every minute. Since the Quatenza patch is transparent for the green light, we were able to estimate parameters of blood circulation and their evolution under the patch.
4. In the last step, the Quatenza patch was removed, and the study area was cleaned by a special gel.

By this way, we obtained a series of video frames for each subject before and after topical application of the capsaicin to the upper arm. Processing of simultaneously recorded video and ECG data allowed us to reveal the parameters of microcirculation in each point of the area under study and their evolution during the experiment.

### Data processing

**Fig. 2:**
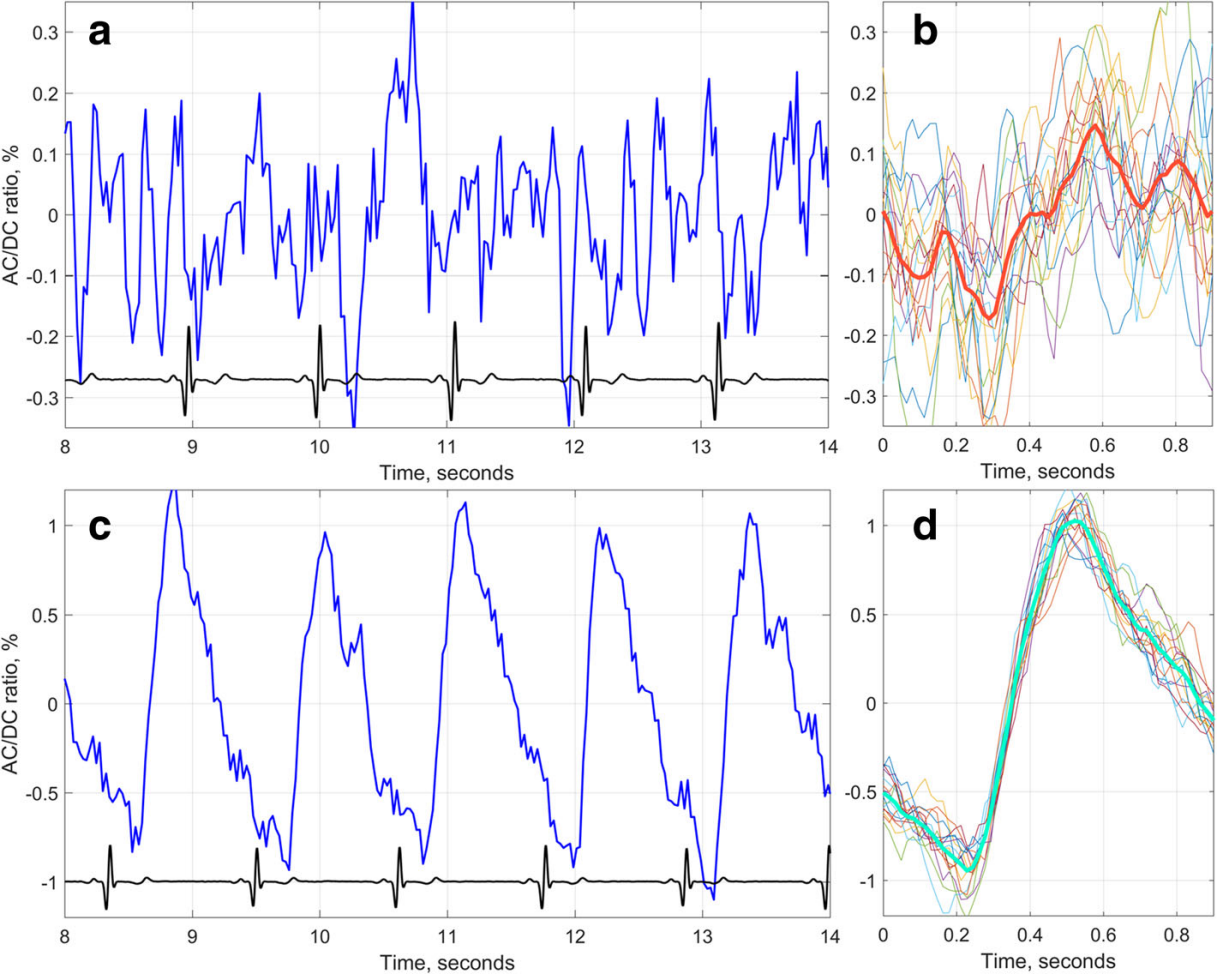
Typical example of PPG waveform measured in the upper arm. Waveform shown in (**a**) was recorded before application of the capsaicin patch, whereas that in (**c**) was recorded at about 15-th minute of the patch application. Black curves in (**a**) and (**c**) show synchronously recorded ECG. Thick lines in (**b**) and (**d**) show the shape of the signal after averaging over 20 cardiac cycles

Each series of recorded video frames was processed off-line by using custom-made software implemented in the MATLAB platform. First, we calculated the spatial distribution of the blood pulsations amplitude (BPA) by using the algorithm described in details in our previous paper [18]. Briefly, the technique includes the following steps. (i) in the recorded image of subject’s upper arm, we manually selected an area slightly larger than the Quatenza patch. It was automatically covered by small non-overlapping regions of interest (ROI) sizing 5 × 5 pixels (1.5 × 1.5 mm^2^ in the arm). (ii) in every ROI we calculated frame-by-frame evolution of average pixel values to evaluate a PPG waveform. Typical waveform consisted of alternative component (AC) modulated at the heartbeat frequency, which was superimposed with the slowly varying component (DC). After calculation of AC-to-DC ratio, deducing the unity, and inverting the sign, we obtained a PPG waveform. Examples of the waveforms are shown in Fig. 2a, c. (iii) we defined the beginning of each cardiac cycle by the position of every R-peak in ECG, summarized all PPG pieces recorded during 20 s to evaluate the mean PPG shape of one cycle (shown in Fig. 2b, d), and calculated blood pulsation amplitude (BPA) as difference between maximal and minimal values of the mean PPG waveform.

**Fig. 3:**
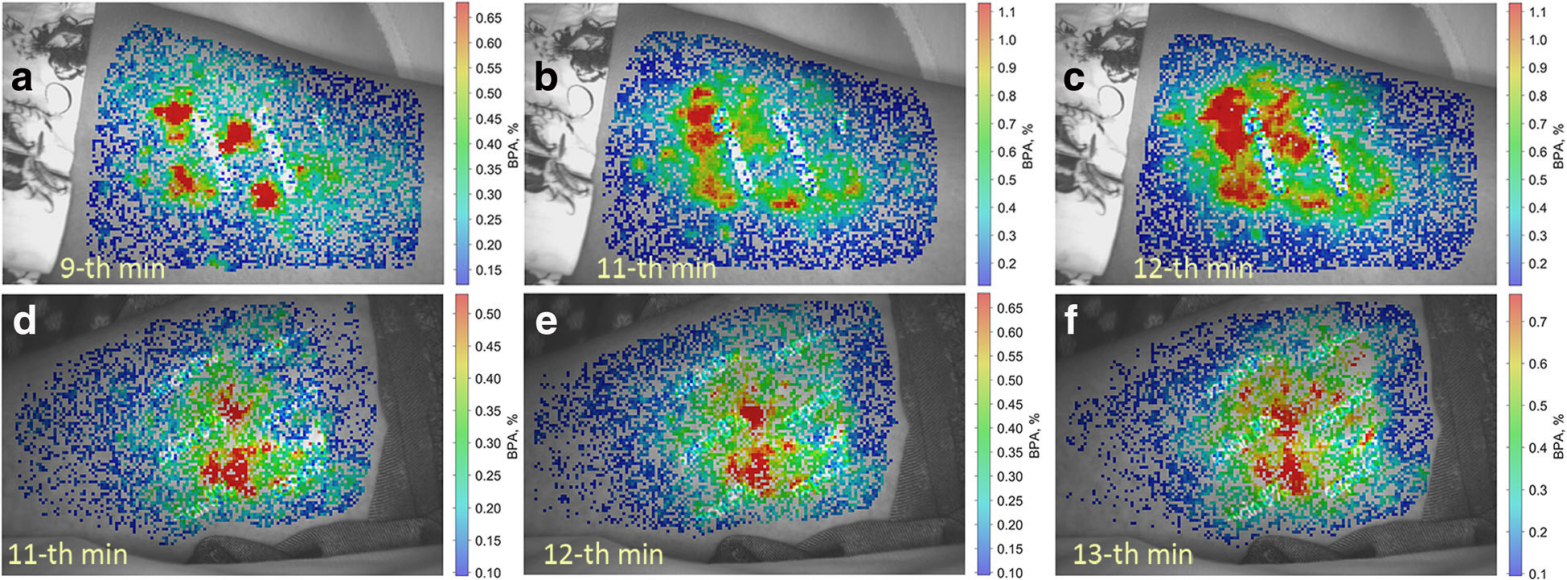
Evolution of the spatial distribution of blood pulsation amplitude in the upper arm during capsaicin application. BPA maps for migraine patients are shown in the upper raw (**a**-**c**) whereas those for healthy subjects are in the lower raw (**d**-**f**). The color scale on the right of each map shows BPA as AC/DC ratio in percent for each distribution, respectively. The moment of PPG recording is shown in the left lower corner of each map with reference to the beginning of capsaicin application

The typical PPG waveform before Quatenza patch application is shown in Fig. 2a. One can see that the signal modulation is of a noise-like type. In addition, the heartbeat related modulation is hidden in the waveform shown in Fig. 2a. It can be revealed after averaging the PPG signal over 20 or more cardiac cycles if the beginning of each cardiac cycle is known (that is achieved by synchronous recording of PPG and EGC). Thick red curve in Fig. 2b shows the mean shape of one cardiac cycle whereas thin colored curves show the signals during every cycle presented in phase with R-peaks of ECG. Mean PPG waveform contains heartbeat related modulation affected by physiological noise [18]. The algorithm of BPA estimation allowed us to calculate the spatial distribution of BPA over subject’s arm. Typical examples of BPA maps are shown in Fig. 3.

### Statistical analysis

Statistical analysis was performed using the STATISTICA 10 software. All continuous variables were expressed as the mean ± standard deviation of the mean (SD). The results of the comparative analysis are graphically represented as the mean, error of the mean, and SD. Non-parametric Mann - Whitney U-test was used to assess the reliability of the differences. One-variance dispersion analysis (ANOVA) with selection of the least number of significant indicators was used to assess the coupled variability of blood flow characteristics in the main and control groups. The diagnostic significance of blood flow parameters in response to capsaicin application was assessed through discriminant analysis using the lowest number of indicators. Significance was assumed when *p* < 0.05.

## Results

### Capsaicin-induced blood perfusion

The baseline of blood perfusion (before lidocaine application) in the capillary bed of the upper arm was at low level for majority of subjects (BPA was less than 0.3%). According to the protocol of our experiment, the lidocaine patch was applied for one hour and after its removal, the 8%-capsaicin patch was applied to the same area of the upper arm. At the beginning of capsaicin patch application the noise-like PPG waveforms (such as shown in Fig. 2a) were observed for all subjects. Similar noise-like signals were observed in the baseline recordings before lidocaine application. However, after certain delay time (DT), the modulation amplitude of PPG waveforms started to grow up. The delay time was individual for each subject with the mean value of 7.6 ± 3.5 min ranging from 1.1 to 13.9 min. The growth of BPA was accompanied by changes in the waveform shape that becomes similar to the classical PPG shape with anacrotic waves following the arterial blood pressure change. A typical example of the PPG waveform with increased BPA is shown in Fig. 2c with the mean shape of this waveform during the cardiac cycle shown in Fig. 2d. It is seen in Fig. 2d that individual signals of different cardiac cycles (thin colored lines) are well synchronized with the heartbeats defined by the R-peaks of ECG.

### Blood perfusion dynamics

The transparency of the capsaicin patch for green light allowed us to measure spatial distribution of BPA an its evolution *continuously* during the whole period of patch application. First, we found that BPA was unevenly increased in all participants forming areas with elevated amplitude (“hot spots”) of blood pulsations. These hot spots were clearly corresponded to the locally increased blood flow in the capillary bed. Examples of the spatial BPA distribution and its variation during capsaicin application are shown in Fig. 3 for two subjects, one from the migraine and another from the control group. Notably, there was a remarkable difference between BPA dynamics in these two subjects. Whereas the position of “hot spots” in migraine patient (Fig. 3a-c) strongly varied (providing an impression of migrating spots), there was a rather stable position of the “hot spots” in the control subject (Fig. 3d-f).

**Fig. 4:**
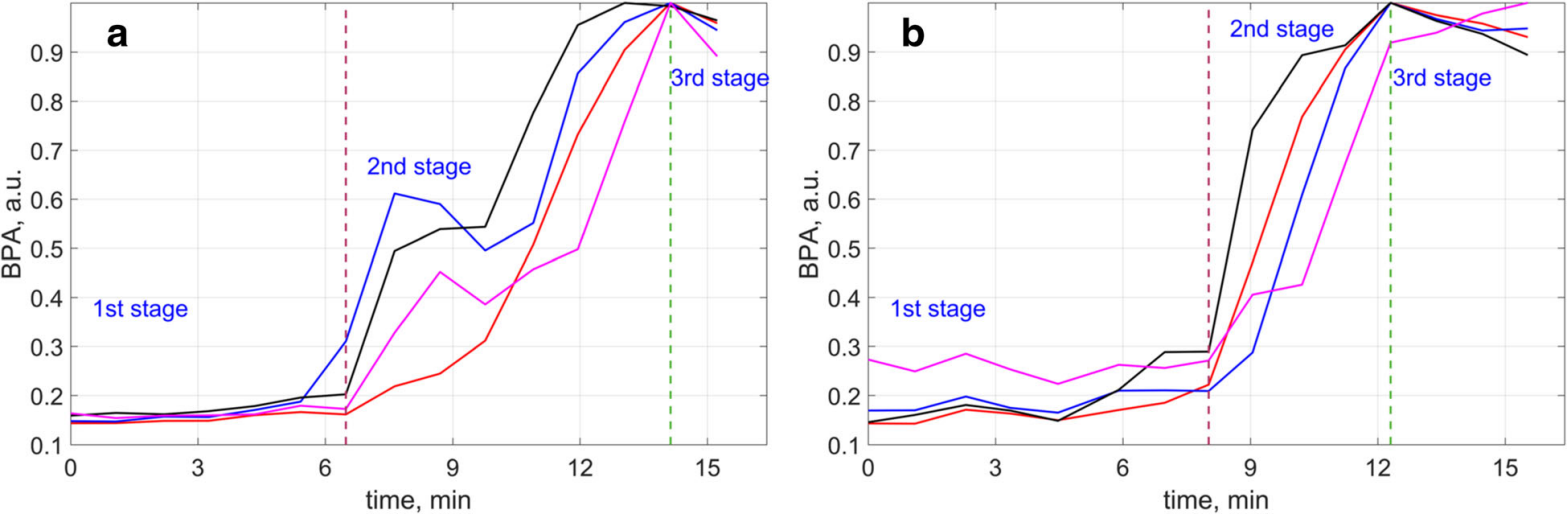
The time course of blood perfusion changes during capsaicin application: an example of BPA evolution in four big ROIs representative for migraine patients (**a**), and an example for healthy subjects (**b**). Vertical dashed lines indicate the moments when the blood perfusion starts to grow (dashed green line) and when it was saturated (dashed red line)

Two representative examples of BPA evolution during capsaicin application (3 for migraine patients and 3 for healthy subjects, respectively) are shown in Fig. 4. In all cases the time-course of BPA changes after capsaicin application consisted of three clearly distinguishable stages. The first stage was represented by a latent period when the heartbeat-related modulation was comparable with noise (see the waveform in Fig. 2b). At the second stage a sharp increase of BPA was observed when the PPG waveform became similar to the waveform of arterial blood pressure (Fig. 2d). At the third stage, there was a saturation of the blood pulsation amplitudes. Each graph in Fig. 4 includes four curves (red, blue, black, and pink) showing BPA evolution in non-overlapping big ROIs placed in the middle of the capsaicin patch. The size of big ROIs was chosen to be 1.6 × 1.6 cm^2^ approximately fitting the size of “hot spots” (Fig. 3). Graphs in Fig. 4a show BPA evolution for migraine patients whereas graphs in Fig.4b are of healthy subjects. One can see that there was almost synchronous increase of the blood perfusion in all ROIs for healthy subjects. In contrast, essential desynchronization of BPA growth was observed in migraine patients. Thus, there were several peaks in the growing phase of capsaicin-induced signal providing the increase of BPA in one ROI to be accompanied by the decrease in other ROI (Fig. 4a). Notably, this is another way of manifestation of the “hot spot” migration depicted in Fig. 3.

**Fig. 5:**
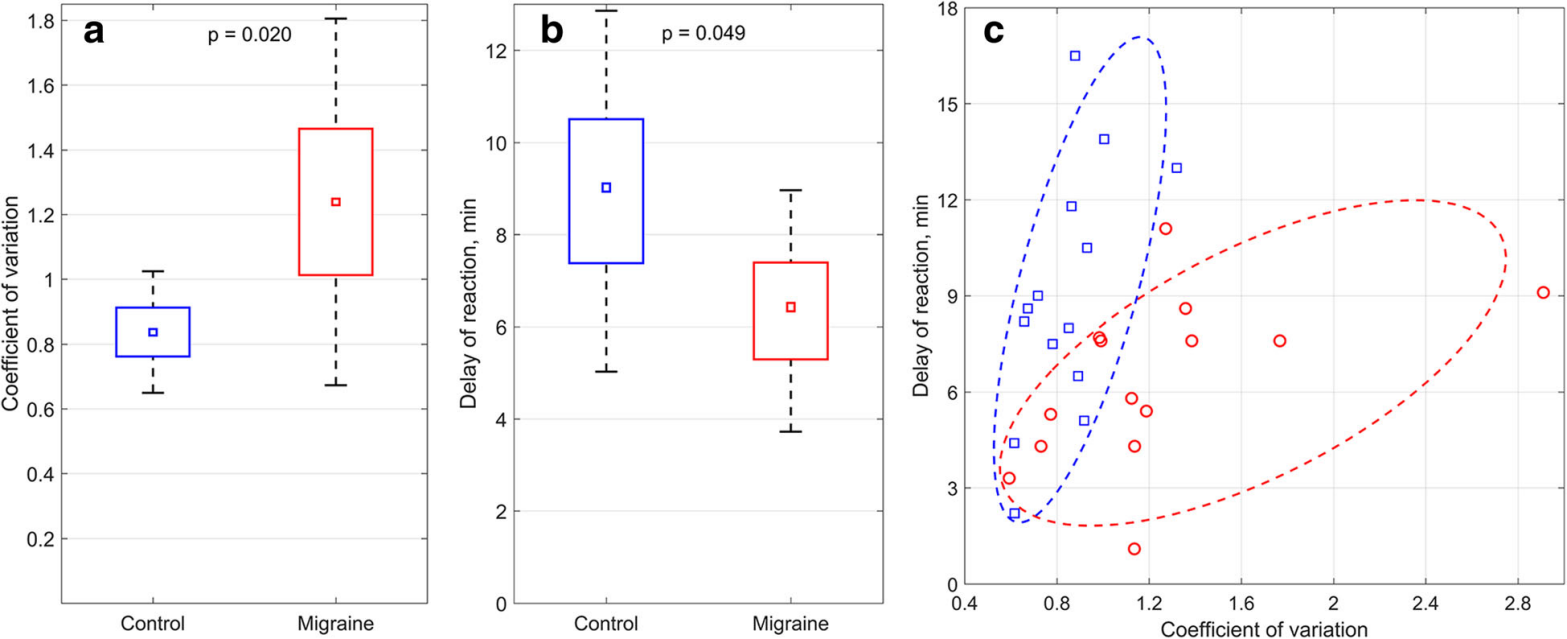
Parameters of capsaicin-induced blood perfusion in control and migraine groups. **a** Coefficient of variation and **b** delay time of capsaicin reaction in migraine patients and control group. Data presented as the mean (small squares), error of mean (boxes), and SD (whiskers). **c** Two-dimensional distribution for CV and DT in the Control and Migraine groups. Red circles are for migraine patients, and blue squares are for healthy subjects. The dashed red line shows the region of the predominant distribution of the parameters for migraine group, whereas the dashed blue line is for the control group

We have also found that the mean speed of the BPA growth during capsaicin application (stage 2) was different for different subjects varying from 0.04 to 0.19% per min. However, this parameter did not differ significantly between migraine and control groups: 0.11 ± 0.04 for migraine, *n* = 14 versus 0.09 ± 0.03 for control, n = 14, *p* = 0.10. For quantitative estimation of the “hot spots” migration we used the coefficient of variation (CV) which is the ratio of the BPA-growth speed averaged over all big ROIs in the time interval between the start of growth and signal saturation (indicated by dashed lines in Fig. 4) and its standard deviation. Comparative analysis showed that the CV value in migraine group (1.24 ± 0.57) is significantly different from that in control group (0.84 ± 0.19, *p* = 0.02, Fig. 5a). This finding demonstrated for the first time essential differences in reactivity of the microcirculation to capsaicin application between migraine and control groups.

Moreover, we found that migraine patients, compared to control group, have a smaller DT of BPA growth: 6.3 ± 2.6 min versus 8.9 ± 3.9 min, *р* = 0.049, Fig. 5b, which served as a basis for studying the joint variability of the parameters CV and DT. Univariant dispersion analysis revealed that joint dispersion of the CV and DT are significantly different in migraine and control groups: *p* = 0.018 with the Fisher’s criterion F = 6.37 for CV, and *p* = 0.049, F = 4.27 for DT. Distribution of CV and DT in both groups is shown in Fig. 5c. It is seen that most of migraine patients are situated within the area limited by the dashed red line. The migraine group is characterized by higher CV and lower DT of larger dispersion. In contrast, healthy subjects possess larger DT and lower CV. These parameters for the control group are located within the area limited by the dashed blue line in Fig. 5c. Discriminant analysis using parameters of CV and DT allowed us to correctly classify 23 (82%) of 28 participants (*p* < 0.005). Furthermore, a joint analysis of variance mean speed and STD also allowed detecting significant differences between patients with migraine and control groups: *p* = 0.026 with the Fisher’s criterion F = 4.23.

### Capsaicin-induced hyperemia

**Fig. 6:**
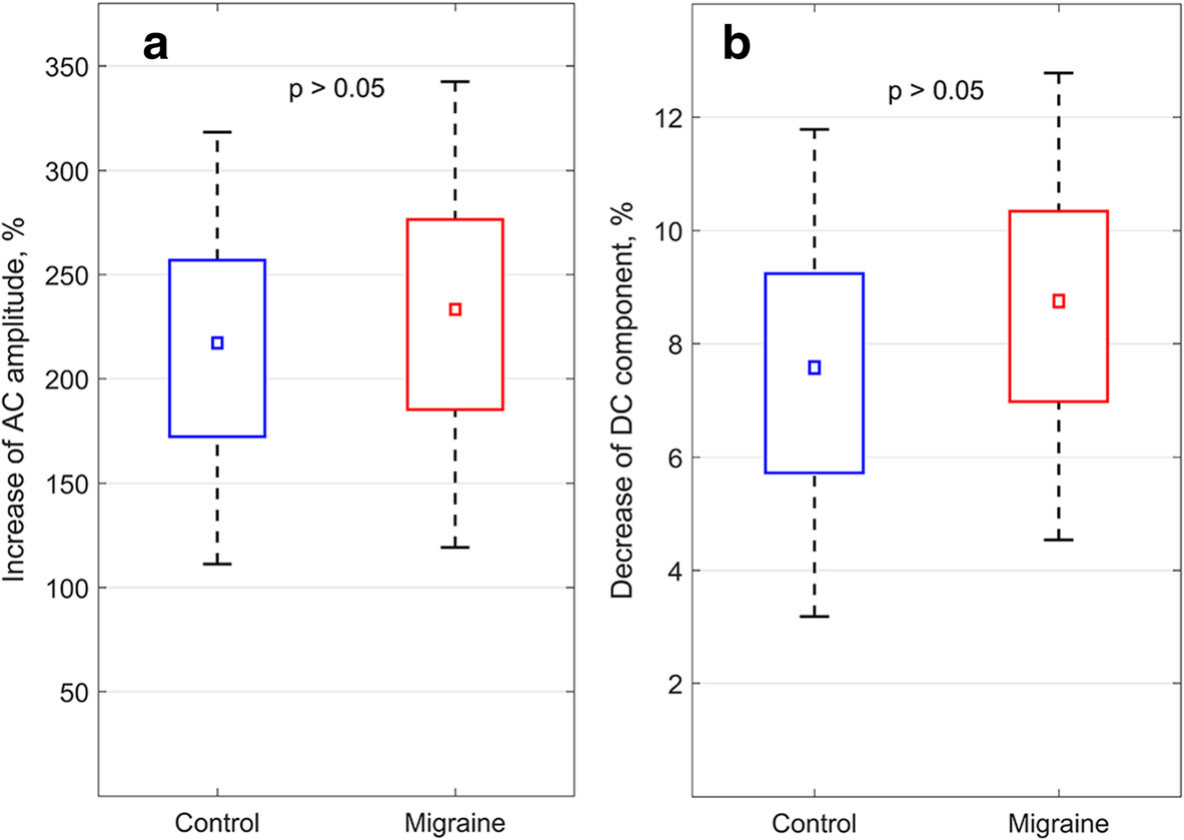
Change of AC (panel **a**) and DC (panel **b**) components of the PPG waveform in control (blue) and migraine (red) groups after capsaicin application. The data are presented as the mean (small squares), error of mean (boxes), and SD (whiskers)

A typical reaction of the human skin to capsaicin application is progressive skin redness [10], which becomes apparent as an increase of green light absorption. Therefore, in our experiments, the skin redness (or hyperemia) could be estimated as a relative change of the DC component of the PPG waveform measured in the place of patch application. To be consistent with estimations of AC-component change, we calculated the DC-component change in the time interval between the beginning of the reaction and signal saturation (indicated by dashed lines in Fig. 4). As shown in Fig. 6, we found that the degree of hyperemia was highly variable among subjects. Nevertheless, in both groups, the changes of the DC component were relatively small as they did not exceed one tenth of the initial value: 8.7 ± 4.1% and 7.8 ± 4.3% for migraine and control group, respectively. In sharp contrast, the much more pronounced increase of AC component was observed both in the control (215 ± 100%) and migraine (231 ± 110%) groups. It means that BPA increase occurs mainly because of change of AC component. No significant difference in either DC or AC change was found between the groups (*p* > 0.05).

Figure 6 shows that skin redness evaluated as changes of the DC component of PPG was twenty-fold less than changes of the amplitude of AC component. Therefore, the reaction on the capsaicin application cannot be assessed by a simple comparison of the skin redness as efficiently as by imaging photoplethysmography.

## Discussion

Based on innovative IPPG technique, we present here the novel method for evaluation of capsaicin induced changes in peripheral blood flow that is more sensitive than the commonly used skin redness analysis. The main finding of our study is the remarkable variability (both in space and time) in skin microcirculation after capsaicin application in migraine patients. We suggest this novel phenomenon can serve as a quantitative biomarker of vascular changes in this disorder and probably as a predictive parameter for efficiency of anti-migraine CGRP-based drugs.

Despite the high prevalence of migraine, the pathogenesis of migraine pain remains unclear. However, recent animal and human studies suggested the significant role of TRPV1 receptors expressed in meningeal nerve fibers in migraine pathology [4, 5]. Accumulated evidence suggests that the neuropeptide CGRP is released upon activation of TRPV1 receptors as the main triggering factor implicated in migraine pain [6–8]. Therefore, the inhibition of the pro-nociceptive action of CGRP represents one of the most promising approaches for migraine treatment [7, 9]. For instance, the most specific agents for migraine treatment are represented by triptans which block CGRP release [21–23]. Another promising approach is neutralization of CGRP (or its receptors) by specific antibodies [24–26]. Although other treatment strategies are possible, the known contribution of CGRP to migraine pathology in the individual patients could predict the efficiency of triptans or CGRP antibodies.

Capsaicin-induced increase in DBF presented as the skin redness is a simple test to evaluate the release of CGRP in peripheral tissues and to test the efficiency of anti-migraine medicines [2]. Measuring the intensity of skin redness potentially could indirectly suggest the sensitivity to CGRP based medications. Consistent with this view, it has been shown recently that migraine patients have larger areas of skin redness after dermal application of capsaicin [10] suggesting the larger release or higher reactivity of vessels to endogenous CGRP which is one of the most powerful vasodilators. Migraine has been suggested as a systemic vasculopathy [27]. Vascular changes in migraine were suggested since the emergence of the famous Woolf’s vascular theory of migraine [28]. This theory proposed the primary changes in the intracranial vessels which are hardly available. Nevertheless, the recent magnetic resonance angiography of extra- and intracranial vessels indicated the dilatation of blood vessels during migraine attack [29]. The other study reported the local asymmetry of forehead blood flow in the affected side of the migraine patient’s face during an attack [30]. However, unlike most of previous studies that have addressed the functional states of large vessels, our study was focused on variability of microcirculation in migraine patients. Here we report ‘*local asynchronous*’ reactions in certain skin areas triggered by application of capsaicin which were higher presented in migraine. One of the main findings of our study is highly heterogeneous (in time and in space) reaction of migraine patients to stimulation of TRPV1 receptors. It became possible to obtain this novel finding through the use of the advanced IPPG technique allowing us to register the pulsatile blood flow with simultaneous ECG recordings, resulting in enhanced sensitivity of this approach [18]. Another important observation is that the essential changes in BPA could develop without significant skin redness (evaluated here via calculation of the DC component of IPPG). Notably, the degree of DC changes in these relatively short period of capsaicin application was much weaker than BPA modulation. Taken together these data suggest that BPA measurement in hot spots and its variability (measured from the coefficient of BPA variations) represent the powerful biomarker of blood flow changes associated with migraine. The nature of migrating hot spots remains unclear but it could be related to the random opening and closure of precapillary sphincters which regulate blood flow in the capillaries and venules. This dynamic process could be determined by interaction of several competing mechanisms including CGRP mediated vasodilation and the opposite process of vasoconstriction based on direct activation of vascular TRPV1 receptors by capsaicin [31, 32]. It is worth noting that the local anesthetic lidocaine was applied before capsaicin due to instructions of the Quatenza plaster manufacturer. Recent study reported that lidocaine can directly stimulate TRPV1 and TRPA1 receptors and release CGRP [33] which potentially can induce vasomotor effects similar to those of capsaicin. However, in our conditions, relatively low concentrations of lidocaine did not produce measurable changes of the microcirculation.

Interestingly, the phenomenon of hot spots leading to asynchronous high intensity activation of local parts of the skin can unexpectedly slow down the global growth of the BPA masking thus the reactivity of the current individual to capsaicin. This can explain why we did not find significant changes in the speed of BPA growth in migraine patients in our small sample. Nevertheless, high variability of BPA in capsaicin test can predict the sensitivity of CGRP-specific treatments such as triptans and CGRP neutralizing antibodies.

Left-right side asymmetry (asynchronous reactions) in the sympathetic skin responses was also found in the headache-free period in unilateral migraine patients [30]. One of the key questions is whether the local reaction represented as migrating hot spots after application of capsaicin to migraine patients was mediated by pure peripheral mechanisms or operated via central sensitization [34]. The latter can potentially change the autonomous control of microcirculation via neuronal mechanisms. Indeed, we found recently that during interictal period in migraine patients a specific enhancement of the sympathetic control is observed [35].

The main limitation of the study is that it was performed in a small cohort and the data should be confirmed in a larger population. However, even in a limited number of patients we found the novel criterion for evaluation of the peripheral blood flow such as the high variability of zones in the skin with increased BPA. In addition, the trend to a shorter latency in BPA increase should be revisited in a larger population. We also used lidocaine as the obligatory component of the pre-treatment that may have partially masked the intensity of capsaicin effects. The genetic reason for asynchronous activation of microcirculation remains unclear. As TRPV1 gene polymorphism determines sensitivity to capsaicin [1] further studies are needed to explore the role of genotyping-based approaches in migraine patients. Suggested here BPA changes with IPPG technique could be used to determine the sensitivity of the particular person to capsaicin as a prognostic tool to identify the role of CGRP component in his/her vascular reactions.

## Conclusion

In conclusion, in this pilot study, we suggest the novel non-invasive biomarkers of migraine such as the BPA-growth-speed variations observed during mild activation of skin TRPV1 receptors with capsaicin. This simple test performed with transparent capsaicin patch could serve for the diagnostic purposes and for the prediction of the personalized treatments of migraine patients.

## Abbreviations

AC: Alternative component
BPA: Blood pulsation amplitude
CGRP: Calcitonin gene related peptide
CMOS: Complementary metal-oxide-semiconductor
CV: Coefficient of variation
DBF: Dermal blood flow
DC: Slowly varying component
DT: Delay time of the microcirculation reaction on the capsaicin application
ECG: Electrocardiogram
ICHD: the International classification of headache disorder
IPPG: Imaging photoplethysmography
LED: Light-emitted diode
NSAID: Non-steroid anti-inflammatory drugs
PNG: Portable network graphics format
PPG: Photoplethysmographic
ROI: Region of interest
SD: Standard deviation
TRPV1: Transient receptor potential vanilloid type 1
